# 5-Hydroxycyclopenicillone, a New β-Amyloid Fibrillization Inhibitor from a Sponge-Derived Fungus *Trichoderma* sp. HPQJ-34

**DOI:** 10.3390/md15080260

**Published:** 2017-08-19

**Authors:** Fang Fang, Jiaying Zhao, Lijian Ding, Chunhui Huang, C. Benjamin Naman, Shan He, Bin Wu, Peng Zhu, Qijun Luo, William H. Gerwick, Xiaojun Yan, Qinwen Wang, Zaijun Zhang, Wei Cui

**Affiliations:** 1 Li Dak Sum Yip Yio Chin Kenneth Li Marine Biopharmaceutical Research Center, Ningbo University, Ningbo 315211, China; 15888107865@163.com (F.F.); 13511319513@163.com (J.Z.); dinglijian@nbu.edu.cn (L.D.); m15757460040@163.com (C.H.); zhupeng@nbu.edu.cn (P.Z.); 2 Center for Marine Biotechnology and Biomedicine, Scripps Institution of Oceanography and Skaggs School of Pharmacy and Pharmaceutical Sciences, University of California, San Diego, La Jolla, CA 92093, USA; bnaman@ucsd.edu (C.B.N.); wgerwick@ucsd.edu (W.H.G.); 3 Key Laboratory of Marine Biotechnology of Zhejiang Province, Ningbo University, Ningbo 315211, China; luoqijun@nbu.edu.cn (Q.L.); yanxiaojun@nbu.edu.cn (X.Y.); 4 Ocean College, Zhejiang University, Hangzhou 310058, China; wubin@zju.edu.cn; 5 School of Medicine, Ningbo University, Ningbo 315211, China; wangqinwen@nbu.edu.cn; 6 Institute of New Drug Research and Guangzhou Key Laboratory of Innovative Chemical Drug Research in Cardio-cerebrovascular Diseases, College of Pharmacy, Jinan University, Guangzhou 510632, China; zaijunzhang@163.com

**Keywords:** sponge-derived fungus, cyclopentenone, *Trichoderma* sp. HPQJ-34, Alzheimer’s disease

## Abstract

A new cyclopentenone, 5-hydroxycyclopeni cillone (**1**), was isolated together with three known compounds, *ar*-turmerone (**2**), citreoisocoumarin (**3**), and 6-*O*-methyl-citreoisocoumarin (**4**), from a culture of the sponge-derived fungus *Trichoderma* sp. HPQJ-34. The structures of **1**–**4** were characterized using comprehensive spectroscopic analyses. The absolute configuration of **1** was determined by comparison of electronic circular dichroism (ECD) spectra with literature values used for the reported analogue, cyclopenicillone (**5**), which was not isolated in this research. Compound **1** was shown to scavenge 2,2-diphenyl-1-picrylhydrazyl free radicals, and decrease β-amyloid (Aβ) fibrillization in vitro. Moreover, **1** significantly reduced H_2_O_2_-induced neurotoxicity in SH-SY5Y cells. These findings suggested that compound **1**, a newly discovered cyclopentenone, has moderate anti-oxidative, anti-Aβ fibrillization properties and neuroprotective effects, and might be a good free radical scavenger.

## 1. Introduction

**Figure 1:**
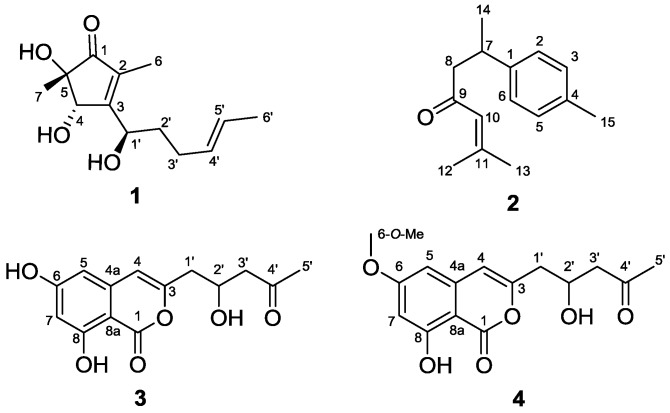
The chemical structures of **1**–**4**, isolated from the sponge-derived fungus *Trichoderma* sp. HPQJ-34.

Marine fungi continue to be an important source for the discovery of structurally diverse secondary metabolites with a wide variety of biological activities [1,2,3,4,5,6,7,8,9,10]. Fungi isolated from sponges are well-known as prolific producers of new natural products, and a considerable number of these have displayed promising biological and pharmacological properties such as antiviral, antibacterial, antitumor, antifouling, anti-inflammatory, as well as immunomodulatory activity [11,12,13,14]. In the search for new pharmaceutical leads in sponge-associated fungi, the organic extract of *Trichoderma* sp. HPQJ-34, isolated from the marine sponge *Hymeniacidon perleve,* was found to have potent anti-oxidative, anti-Aβ fibrillization properties and neuroprotective effects during preliminary testing. Bioactivity-guided chromatographic separation of the EtOAc extract obtained from the fermentation broth of this strain resulted in the isolation of a new cyclopentenone, 5-hydroxycyclopenicillone (**1**), along with three known compounds: *ar*-turmerone (**2**) [15], citreoisocoumarin (**3**) [16] and 6-*O*-methyl-citreoisocoumarin (**4**) [17] (Figure 1).The details of the isolation, the structure identification or elucidation, and the biological activities associated with these fungal metabolites are reported below.

## 2. Results and Discussion

### 2.1. Isolation and Taxonomy of the Producing Microorganism

**Figure 2:**
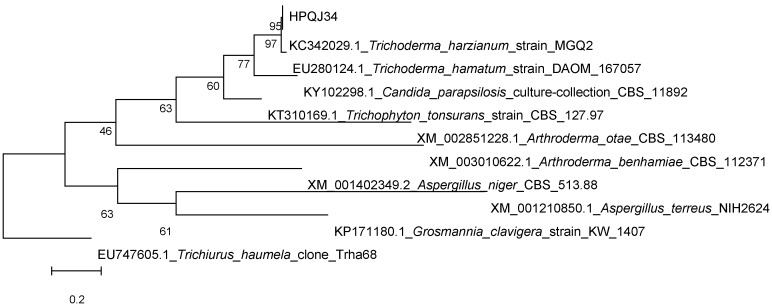
Phylogenetic tree built with Molecular Evolutionary Genetics Analysis (MEGA) 5.05 based on nearly complete ITS rDNA gene sequences of HPQJ-34.

The fungal strain *Trichoderma* sp. HPQJ-34 was isolated from the sponge *Hymeniacidon perleve* collected from Dongji Island, Zhejiang, China. It was identified as *Trichoderma* sp. HPQJ-34 according to morphological and molecular (ITS rDNA sequence) analyses. A BLASTN search employing the Polymerase Chain Reaction (PCR)-amplified Internal Transcribed Spacer (ITS) rDNA sequence (640 bp) indicated that the strain was closely related to *Trichoderma harzianum* strain MGQ2 (T) (99% similarity). A phylogenetic tree was constructed using the neighbor-joining method corrected with the Jukes–Cantor algorithm (Figure 2), also showing that the HPQJ-34 strain is a member of the genus *Trichoderma* [18].

### 2.2. Structure Elucidation

**Figure 3:**
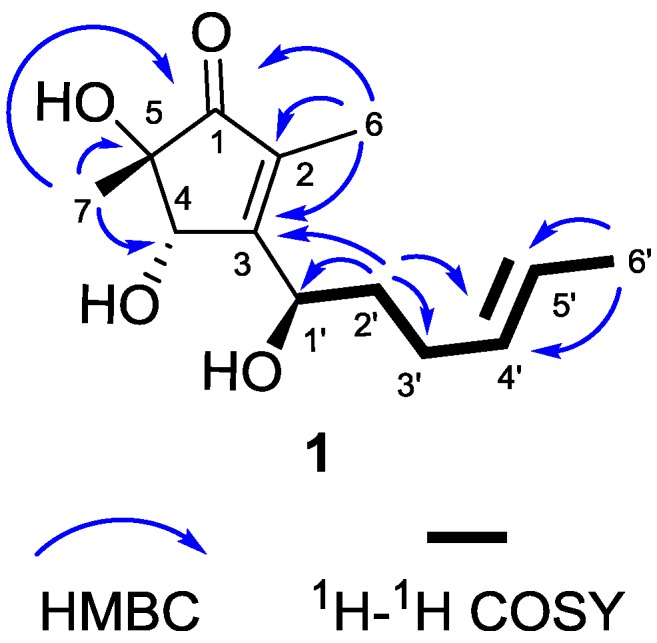
Selected HMBC and ^1^H–^1^H COSY correlations observed for **1**.

The new molecule, 5-hydroxycyclopenicillone (**1**), was obtained as a yellow oil. Compound **1** has the molecular formula C_13_H_20_O_4_, which was determined from a sodiated molecular ion adduct peak in the High-resolution electrospray ionisation mass spectrometry (HRESIMS) at m/z 263.1247 [M + Na]^+^ (calcd. for C_13_H_20_O_4_Na^+^, 263.1254). The ^1^H NMR spectrum of **1** in CD_3_OD showed two methyl doublets at δ_H_ 1.65 (3H, d, J = 4.8 Hz, CH_3_-6′) and 1.75 (3H, d, J = 1.0 Hz, CH_3_-6), one methyl singlet at δ_H_ 1.23 (3H, s, CH_3_-7), two olefinic methines at δ_H_ 5.49 (1H, m, CH-4′) and 5.48 (1H, m, CH-5′), two oxygenated methines at δ_H_ 4.49 (1H, d, J = 1.0 Hz, CH-4) and 4.72 (1H, dd, J = 8.6, 4.8 Hz, CH-1′), as well as signals with complex coupling patterns attributed to two methylenes between δ_H_ 1.76 and 2.12 (Table 1). The four degrees of unsaturation inherent in the molecular formula of **1**, together with data showing the presence of one carbonyl and four olefinic carbons in the ^13^C NMR and Distortionless Enhancement by Polarization Transfer (DEPT) spectra, indicated that the structure of **1** possesses one ring [19]. Selected Heteronuclear Multiple Bond Correlation (HMBC) correlations, shown in Figure 3, including from CH_3_-7 to C-1, C-4 and C-5, from CH_3_-6 to C-1, C-2, and C-3, and from CH-4 to C-3 indicated the presence of a 3-substituted 4,5-dihydroxy-2,5-dimethylcyclopent-2-enone ring system in **1**. This assignment was further supported by the homoallylic coupling (J = 1.0 Hz) observed between CH-4 and CH_3_-6. Consecutive ^1^H–^1^H COrrelated SpectroscopY (COSY) correlations from CH_3_-6′ to CH-5′, then CH-4′, CH-3′, CH-2′ and finally CH-1′ suggested a 1-disubstituted 1-hydroxyhex-4-ene moiety as an additional substructure of **1**. This was reinforced by HMBC correlations observed from CH_3_-6′ to C-4′ and C-5′, as well as from CH-2′ to C-1′, C-3′, and C-4′. Finally, the connection between the two structural fragments was established as a C-3 to C-1′ linkage by the HMBC correlation observed from CH-2′ to C-3, thus completing the planar structure of **1**. 

**Figure 4:**
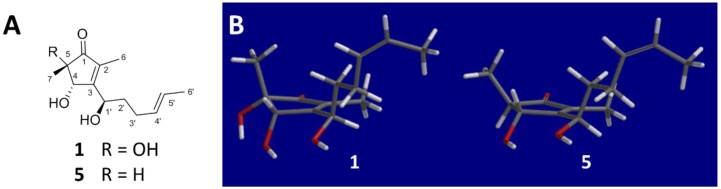
Structural comparison of **1** with reported analogue **5**. Panel A: Chemical structures of **1** and **5**. Panel B: Energy-minimized computational molecular models of **1** and **5**, showing a similar conformation.

Because the ^1^H NMR signals for CH-4′ and CH-5′ in **1** significantly overlapped, *J* coupling analysis was precluded. Therefore, the double bond connecting these carbons was assigned as *trans* based on the deshielded ^13^C NMR shift of the terminal methyl carbon, C-6ʹ (*δ_C_* 18.1). If the C-4′ to C-5′ double bond was *cis*-configured, this terminal methyl carbon would be significantly more shielded, in the range of approximately 10–13 ppm [19]. The NMR data for this terminus of **1**, and in fact the entire 1-hydroxyhex-4-ene moiety, closely matched the reported values for the structural analogue cyclopenicillone (**5**), which was not isolated in this study [19]. In fact, the NMR data for **1** and **5** were remarkably similar (Supplementary Materials Table S11), with obvious differences including the loss of a *J-*coupling partner for CH-4 (a sharp singlet in **1** and a broad singlet in **5**), accompanied by proximal deshielding effects on C-5 (shifted to *δ_C_* 74.5 in **1** from 50.9 in **5**) and C-7 (shifted to *δ_C_* 23.1 in **1** from 13.8 in **5**) [19]. These observations further supported the structure assignment of **1**, and strongly suggested a shared relative, or possibly absolute, configuration between the two structural analogues (Figure 4, panel A), because the NMR parameter of chemical shift (*δ*), especially for the ^13^C nucleus, is particularly sensitive to perturbations in conformation and differences in configuration.

It was also observed that, for **1**, as previously reported for **5 [19]**, the NOE between CH_3_-6 and CH-1ʹ along with CH-4 and CH-2ʹ indicated a restricted free rotation about the C-3 to C-1′ bond. This is likely due to a combination of steric hindrance resulting from the relative inflexibility of the cyclopentenone ring, along with an intramolecular hydrogen bond. The latter was indicated by a broad IR stretch at 3369 cm^−1^ in the spectrum of **1**, as was previously described for **5** [19]. Both OH-1′ and OH-4 are expected to exhibit hydrogen bonding in **1** and **5**, however, OH-5 may also interact with OH-4 in the case of **1**. To examine this further, an energy minimized computational molecular model was generated for both **1** and **5**, using the Hartree–Fock method, to visualize their preferred conformations (Figure 4, panel B). Because both the molecular modeling and NMR data collected for **1** and **5** strongly suggest a shared conformation for these two molecules, and because the absolute configuration of **5** was previously characterized, including the use of theoretically predicted ECD spectra for its low energy molecular modeling conformers, it was reasoned that an electronic circular dichroism (ECD) spectrum could be used to resolve the absolute configuration of **1**.

**Figure 5:**
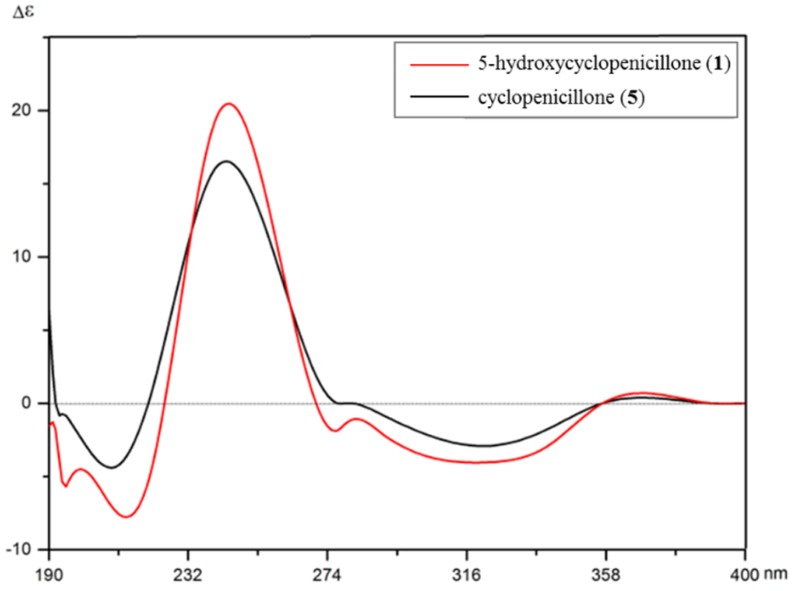
The electronic circular dichroism (ECD) spectra of 5-hydroxycyclopenicillone (**1**) and cyclopenicillone (**5**); from reference [19].

The use of ECD has been well established as a powerful tool for assigning the absolute configuration of natural products [20,21,22]. In the experimental (ECD) spectrum of **1**, the local maxima of the positive Cotton effect (CE) was observed at 246 nm, and negative CEs were observed around 321 and 214 nm. By comparison, the experimental ECD spectrum of **5** was similar to that of **1**, with obvious positive CE maxima at 240 nm accompanied by negative CEs around 326 and 209 nm (Figure 5) [19]. In both cases, the observed positive Davydov splitting for positive first, negative second CEs can be attributed to a positive angle of rotation present between the enone and alkene chromophores, as also suggested by the molecular models presented in Figure 4. As a result, the absolute configuration of **1** was determined to match the reported configuration of **5**, or 4(S), 5(S),1′ (R). Consequently, the structure of **1** was unambiguously established and named as 5-hydroxycyclopenicillone (**1**) {IUPAC: (4S,5S)-4,5-dihydroxy-3-[(R,E)-1-hydroxyhex-4-en-1-yl]-2,5-dimethylcyclopent-2-en-1-one}.

In addition to **1**, three known metabolites were isolated during the course of this research. These were identified, by comparing their spectrometric and spectroscopic data with literature values, as *ar*-turmerone (**2**) [15], citreoisocoumarin (**3**) [16], and 6-*O*-methyl-citreoisocoumarin (**4**) [17]. 

### 2.3. Biological Activities

**Figure 6:**
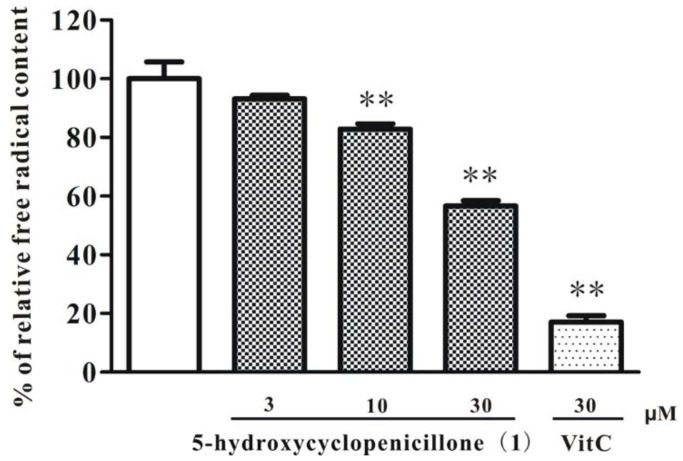
5-hydroxycyclopenicillone (**1**) scavenges DPPH free radicals in a concentration-dependent manner. Compound **1** or vitamin C (VitC) at indicated concentrations were added to 0.2 mM DPPH solution for 20 min. The concentration of DPPH free radicals was measured by evaluating the absorbance at 517 nm. Data are presented as the mean ± SEM of three separate experiments; ** *p* < 0.01 vs. the control group (ANOVA and Dunnett’s test).

The effects of 5-hydroxycyclopenicillone (**1**) were evaluated for the scavenging of free radicals. As shown in Figure 6, compound **1** concentration–dependency reduced the content of DPPH free radicals, suggesting that it might act as a moderate free radical scavenger. These anti-oxidative effects of **1** might be due to the presence of multiple hydroxy groups. Many anti-oxidants, such as vitamin C and vitamin E, are useful for the delay of onset and relief of symptoms of Alzheimer’s disease (AD) [23]. Therefore, it was hypothesized that **1**, a new cyclopentenone with moderate free radical scavenging properties, might be effective in AD models.

**Figure 7:**
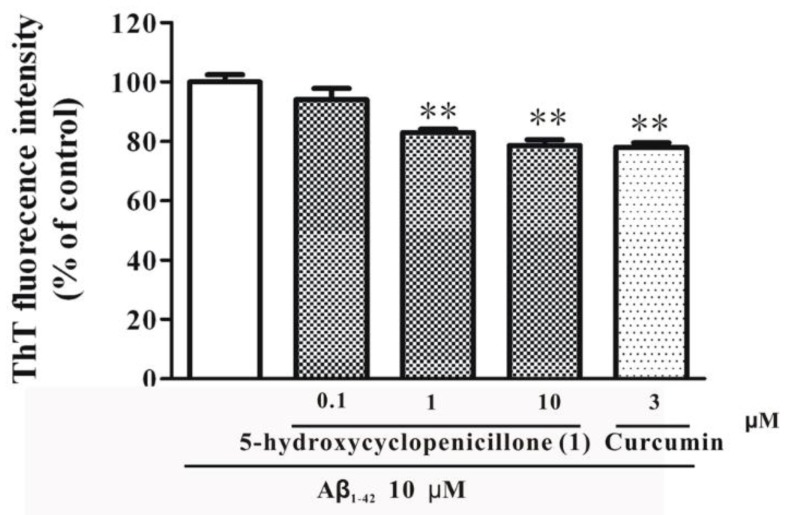
5-Hydroxycyclopenicillone (**1**) significantly decreases the formation of Aβ_1-42_ fibrils. Aβ_1-42_ monomers (10 μM) were incubated with or without **1** or curcumin at indicated concentrations for 3 days. Aβ fibrils were measured using the thioflavin T (ThT) assay. ** *p* < 0.01 vs. Aβ_1-42_ group (ANOVA and Dunnett’s test).

Previous studies have shown that Aβ peptides accumulate in the brain of AD patients [24]. Aβ peptides can form highly toxic oligomers and fibrils, which are considered as the key pathogenic factors of AD [25]. Aβ oligomers and fibrils can further aggregate into plaques, the hallmark of AD. Therefore, drugs that could inhibit the formation of Aβ fibrillization might produce disease-modifying effects for AD treatment [26]. Thus, the effect of **1** on the formation of Aβ fibrils was measured. As shown in Figure 7, co-incubation of **1** and Aβ monomers led to a decreased formation of Aβ fibrils when compared to the incubation of Aβ monomers alone. Moreover, curcumin, a positive control, also inhibited the formation of Aβ fibrils at about the same level as compound **1** (Figure 7). These results suggested that 5-hydroxycyclopenicillone (**1**) could effectively inhibit the formation of Aβ fibrils.

**Figure 8:**
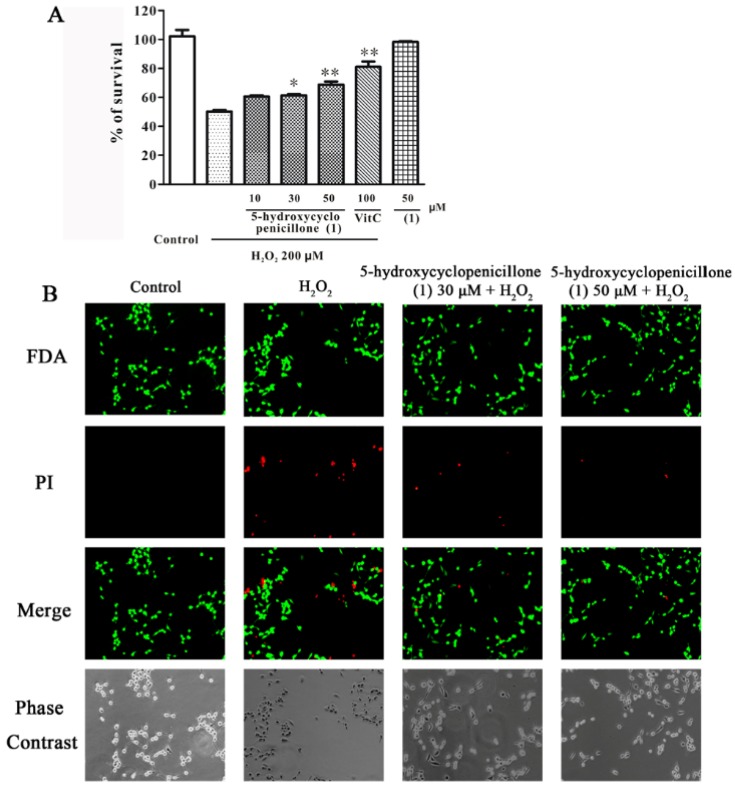
5-hydroxycyclopenicillone (**1**) protects against H_2_O_2_-induced neuronal death in a concentration-dependent manner in SH-SY5Y cells. (A): SH-SY5Y cells were treated with **1** or vitamin C (VitC) at indicated concentrations. After 2 h, cells were exposed to 200 μM of 30% H_2_O_2_ (in H_2_O). MTT assay was used to measure cell viability after 24 h of H_2_O_2_ exposure. Data are expressed as the percentage of control and are presented as the mean ± SEM of three separate experiments; * *p* < 0.05 and ** *p* < 0.01 vs. H_2_O_2_-challenged group (ANOVA and Tukey’s test). (B): SH-SY5Y cells were administrated with **1** for 2 h, and exposed to 200 μM H_2_O_2_. After 24 h, cells were examined by Fluorescein diacetate (FDA)/ propidium iodide (PI) double staining.

Besides Aβ fibrils, oxidative stress also plays an important role in AD pathogenesis [27]. Hydrogen peroxide (H_2_O_2_), an agent widely used to establish oxidative stress-induced neurotoxicity models, can increase intracellular reactive oxygen species and promote neurotoxicity via its acting on various macromolecules [28]. Therefore, the neuroprotective effects of 5-hydroxycyclopenicillone (**1**) on H_2_O_2_–induced neuronal loss in SH-SY5Y cells was investigated. SH-SY5Y cells were pre-treated with **1** or vitamin C for 2 h, followed by treatment with 200 μM of 30% H_2_O_2_ (in H_2_O) for 24 h. Cell viability was determined by the MTT assay. In Figure 8, panel A shows that **1** significantly reduced H_2_O_2–_induced neuronal death in a concentration-dependent manner, demonstrating that 5-hydroxycyclopenicillone is moderately neuroprotectant. Moreover, treatment with 50 μM **1** alone for 26 h was not cytotoxic to cells, suggesting that this agent may be quite safe. Fluorescein diacetate (FDA)/ propidium iodide (PI) double staining for both live cells and dead cells further confirmed that **1** at 30 and 50 μM dramatically protected against H_2_O_2_–induced neuronal death in SH-SY5Y cells (Figure 8, panel B).

## 3. Experimental Section

### 3.1. General Experimental Procedures

Optical rotations were measured on a P-2000 digital polarimeter (JASCO, Hachioji, Japan). The Circular Dichroism (CD) spectra were recorded on a J-1500 spectrophotometer (JASCO, Hachioji, Japan) with a 1 cm path length, 390–190 nm range, 1.00 nm bandwidth, and at a rate of 100 nm/min. UV spectra were recorded on a NADE Evolution 201 spectrophotometer (ThermoFisher, Waltham, MA, USA). IR spectra were acquired on a Nicolet iS5 IR spectrometer (ThermoFisher, Waltham, MA, USA). Also, 1D and 2D NMR spectra were obtained at 500 MHz for ^1^H NMR and 125 MHz for ^13^C NMR on a Bruker AVANCE-500 spectrometer (Bruker, Fällanden, Switzerland). Chemical shifts (*δ*) are referenced to the residual solvent peaks of CD_3_OD (*δ*_H_ 3.31 and *δ*_C_ 49.0) and given in ppm, with coupling constants (*J*) given in hertz (Hz). HRESIMS data were measured using a Q-TOF Premier Mass spectrometer (Waters, Milford, MA, USA). Vacuum liquid chromatography (VLC) was carried out with silica gel (200–300 mesh, Qingdao Marine Chemical Inc. Qingdao, China). Semi-preparative HPLC was performed on a Waters HPLC instrument equipped with a Waters RID-10A detector and a C_18_ column (250 mm × 20 mm ID, 5 µm; YMC Co. Ltd., Tokyo, Japan) by eluting with mixtures of CH_3_OH and H_2_O.

### 3.2. Fungal Material and Fermentation

The fungal strain, *Trichoderma* sp. HPQJ-34, was isolated from the sponge *Hymeniacidon perleve* collected at Dongji Island, Zhejiang, China. It was identified as *Trichoderma* sp. according to morphological and molecular (ITS rDNA sequence) analyses, and was stored in the China General Microbiological Culture Collection Center (CGMCC, No. 12969).

The fungal strain was maintained on slants of modified potato dextrose agar (PDA) medium (potato extract 8.0 g, glucose 20 g, distilled water 1 L, crystal sea marinemix 35 g, agar 20 g; autoclaved at 120 °C for 30 min) at 4 °C. Seed cultures were performed in Erlenmeyer flasks (250 mL) containing 100 mL of Potato Dextrose Broth (PDB) liquid medium (potato extract 8.0 g, glucose 20 g, distilled water 1 L, crystal sea marinemix 35 g) on a shaker at 150 rpm at 25 °C for 3 days. After that, 5 mL seed cultures were inoculated into each 1000 mL flask with 400 mL of medium and cultivated for 14 days (150 rpm, 25 °C).

### 3.3. Extraction and Isolation

The cultures (100 L) were filtered under reduced pressure to produce the filtrate and mycelia. The filtrate was extracted by EtOAc successively (3 × 100 L) to provide the crude extract (40.0 g), which was subjected to vacuum liquid chromatography (VLC) using a stepwise gradient to generate 11 fractions based on the eluting solvent ratio of petroleum ether (PE) to EtOAc (Fr.1, 1:0. Fr.2, 9:1. Fr.3, 4:1. Fr.4, 7:3. Fr.5, 3:2. Fr.6, 1:1. Fr.7, 2:3. Fr.8, 3:7. Fr.9, 1:4. Fr. 10, 1:9. Fr.11, 0:1).

Fraction 2 (2.51 g) was initially subjected to gel filtration on a Sephadex LH-20 column, eluted with CH_3_OH, to produce six subfractions (Fr.2.1–Fr.2.6). Purification of **2** was performed using Fr.2.2 (185.3 mg) and semi-preparative RP-HPLC with CH_3_OH/H_2_O (50:50, *v*/*v*) at 2 mL/min to obtain 10.3 mg (*t*_R_ 28.9 min; 0.026% purification yield from dry EtOAc extract). Fraction 6 (3.75 g) was also separated by Sephadex LH-20 gel filtration chromatography, eluted with CH_3_OH, to give eight subfractions (Fr.6.1–Fr.6.8). Fr.6.5 (55.3 mg) was further separated by semi-preparative RP-HPLC with CH_3_OH/H_2_O (70:30, *v*/*v*) at 2 mL/min to produce **1** (*t*_R_ 40.90 min; 5.3 mg, 0.013% purification yield from dry EtOAc extract). Compound **4** was purified from Fr.6.7 (79.2 mg) by semi-preparative RP-HPLC, applying CH_3_OH/H_2_O (80:20, *v*/*v*) at 2 mL/min (*t*_R_ 21.8 min, 2.4 mg, 0.006% purification yield from dry EtOAc extract). Fraction 7 (1.66 g) was subjected to a Sephadex LH-20 column and eluted with CH_3_OH to produce six subfractions (Fr.7.1–Fr.7.6). Fr.7.1 (200.5 mg) was separated by semi-preparative RP-HPLC with CH_3_OH/H_2_O (85:15, v/v) at 2 mL/min to isolate **3** (*t*_R_ 30.90 min, 15.7 mg, 0.039% purification yield from dry EtOAc extract).

5-hydroxycyclopenicillone (**1**): Yellow oil; [α]${}_{D}^{20}$ = +9.66 (*c* 0.1, MeOH); CD (MeOH) λ_max_ (*Δ*ε) 321 (−4.1), 246 (+21.5), 214 (−8.7) nm; IR (film) *ν*_max_: 3369, 2926, 1710, 1634, 1379, 1055 cm^–1^; UV (MeOH) *λ*_max_ (log ε) 320 (0.7), 238 (3.8) nm; HRESIMS: *m*/*z* 263.1247 [M + Na]^+^ (calcd. for C_14_H_22_O_4_Na^+^, 263.1254); The purity of compound **1** is 98%. ^1^H-NMR (500 MHz, CD_3_OD) *δ*_H_ 5.49 (1H, m, CH-4ʹ), 5.48 (1H, m, CH-5ʹ), 4.72 (1H, dd, *J* = 8.6, 4.9 Hz, CH-1ʹ), 4.49 (1H, d, *J* = 1.0 Hz, CH-4), 2.12 (2H, m, CH_2_-3ʹ), [1.86 (2H, dtd, J = 14.2, 8.6, 5.5 Hz) and 1.76 (1H, m), CH2-2ʹ], 1.75 (3H, d, *J* = 1.0 Hz, CH_3_-6), 1.64 (3H, d, *J* = 4.8 Hz, CH_3_-6ʹ), 1.23 (3H, s, CH_3_-7); ^13^C-NMR (500 MHz, CD_3_OD) *δ*_C_ 210.4 (C-1), 171.7 (C-3), 135.8 (C-2), 131.6 (C-4ʹ), 126.7 (C-5ʹ), 75.3 (C-4), 74.5 (C-5), 69.4 (C-1ʹ), 36.6 (C-2ʹ), 29.8 (C-3ʹ), 23.1 (C-7), 18.1 (C-6ʹ), 8.4 (C-6).

*Ar*-turmerone (**2**): White powder; UV (MeOH) *λ*_max_ (log ε) 290 (3.84), 262 (4.03) nm; IR (film) *ν*_max_: 3363, 2925, 2854, 1726, 1660, 1615, 1381, 1287, 1121, 1074, 976, 543 cm^−^^1^; HRESIMS: *m*/*z* 239.1126 [M + Na]^+^ (calcd. for C_1__5_H_2__0_ONa^+^, 239.1412); The purity of compound **2** is 97%. ^1^H-NMR (500 MHz, CD_3_OD) *δ*_H_ 7.07 (1H, s, CH-2), 7.07 (1H, s, CH-3), 7.07 (1H, s, CH-5), 7.07 (1H, s, CH-6), 6.12 (1H, s, CH-10), 3.21 (1H, m, CH-7), 2.66 (2H, qd, *J* = 15.2, 7.4 Hz, CH_2_-8), 2.27 (3H, s, CH_3_-15), 2.04 (3H, s, CH_3_-12), 1.86 (3H, s, CH_3_-13), 1.21 (3H, d, *J* = 6.9 Hz, CH_3_-14); ^13^C-NMR (500 MHz, CD_3_OD) *δ*_C_ 202.6 (C-9), 157.0 (C-11), 144.6 (C-1), 136.7 (C-4), 130.0 (C-3), 130.0 (C-5), 127.7 (C-2), 127.7 (C-6), 125.2 (C-10), 53.5 (C-8), 37.1 (C-7), 27.6 (C-13), 22.6 (C-14), 21.0 (C-15), 20.8 (C-12).

Citreoisocoumarin (**3**): Yellow oil; UV (MeOH) *λ*_max_ (log ε) 323 (3.47), 293 (3.36) nm; IR (film) *ν*_max_: 3327, 1681, 1625, 1460, 1361, 1238, 1168, 1069, 968, 853, 695, 422, 408 cm^–1^; HRESIMS: 301.0539 [M + Na]^+^ (calcd. for C_1__4_H_14_ONa^+^, 301.0688); The purity of compound **3** is 96%. ^1^H-NMR (500 MHz, CD_3_OD) *δ*_H_ 6.34 (1H, s CH-5), 6.26 (1H, s, CH-7), 6.26 (1H, s, CH-4), 4.40 (1H, dt, *J* = 12.1, 6.4 Hz, CH-2ʹ), 2.64 (2H, dd, *J* = 13.5, 5.4 Hz, CH_2_-3ʹ), 2.55 (2H, dd, *J* = 14.3, 8.0 Hz, CH_2_-1ʹ), 2.14 (3H, s, CH_3_-5ʹ); ^13^C-NMR (500 MHz, CD_3_OD) *δ*_C_ 209.8 (C-4ʹ), 167.8 (C-1), 167.3 (C-6), 164.8 (C-8), 155.6 (C-3), 141.1 (C-4a), 107.4 (C-4), 103.8 (C-8a), 102.7 (C-5), 99.9 (C-7), 66.4 (C-2ʹ), 51.1 (C-3ʹ), 41.9 (C-1ʹ), 30.7 (C-5ʹ).

6-*O*-methyl-citreoisocoumarin (**4**): Yellow oil; UV (MeOH) *λ*_max_ (log ε) 330 (2.88), 290 (2.88) nm; IR (film) *ν*_max_: 3391, 2924, 1684, 1622, 1571, 1509, 1438, 1380, 1241, 1195, 1164, 1074, 1034, 978, 850, 799, 693, 575, 443, 433, 423 cm^–1^; HRESIMS: 315.0721 [M + Na]^+^ (calcd. for C_1__5_H_16_O_6_Na^+^, 315.0845); The purity of compound **4** is 96%. ^1^H-NMR (500 MHz, CD_3_OD) *δ*_H_ 6.50 (1H, d, *J* = 2.3 Hz, CH-5), 6.50 (1H, d, *J* = 2.3 Hz, CH-7), 6.47 (1H, d, *J* = 2.3 Hz, CH-4), 4.46 (1H, m, CH-2ʹ), 3.88 (3H, s, CH_3_-6-*O*-Me), 2.70 (2H, dd, *J* = 8.6, 5.6 Hz, CH_2_-3ʹ), 2.62 (2H, dd, *J* = 14.5, 8.0 Hz, CH_2_-1ʹ), 2.19 (3H, s, CH_3_-5ʹ); ^13^C-NMR (500 MHz, CD_3_OD) *δ*_C_ 209.9 (C-4ʹ), 168.6 (C-6), 166.6 (C-1), 164.1 (C-8), 155.9 (C-3), 141.1 (C-4a), 107.4 (C-4), 102.2 (C-5), 101.6 (C-7), 100.9 (C-8a), 66.4 (C-2ʹ), 56.4 (C-6-*O*-Me), 51.1 (C-3ʹ), 41.9 (C-1ʹ), 30.7 (C-5ʹ).

### 3.4. Computational Molecular Modeling

The energy minimized structures of 5-hydroxycyclopenicillone (**1**) and cyclopenicillone (**5**) were generated using Spartan ′10 software (Wavefunction, Inc., Irvine, CA, USA). An equilibrium geometry calculation was performed for each molecule, at the ground state, using the Hartree–Fock method and the 6-31G* basis set, in a simulated vacuum. All computational operations were completed using an HP Elitebook 850 G1 laptop running 64-bit Windows 7 OS, containing 8 GB ram, and with an Intel i7-4600U CPU @ 2.1 GHz.

### 3.5. Biological Activity Testing of ***1***

The anti-oxidant property of 5-hydroxycyclopenicillone (**1**) was determined by DPPH free radical scavenging assay, as previously reported [29]. Briefly, various concentrations of **1** were added to the methanolic solution of DPPH (0.2 mM). The mixture (200 μL/well) was added to 96-well plates, shaken and kept for 20 min at room temperature. The absorbance was measured at 517 nm by a microplate reader. Vitamin C was used as the positive control. The DPPH free radical scavenging activity was calculated by using the following equation: DPPH free radical scavenging activity (%) = [(absorbance of the control − absorbance of the sample)/absorbance of the control] × 100.

Aβ fibrillization was analyzed by Thioflavin T (ThT) assay as previously reported [30]. Briefly, 10 μM monomeric Aβ_1-42_ was mixed with various concentrations of experimental agents along with 5 μM ThT and the mixture was incubated for 3 days at 37 °C. The fluorescence intensity of the samples was measured by a microplate reader with 440 nm as the excitation wavelength and 485 nm as the emission wavelength.

SH-SY5Y cells were obtained from the Shanghai Institute of Cell Biology (Chinese Academy of Sciences) and maintained in high glucose modified Eagle’s medium (DMEM) supplemented with 10% fetal bovine serum (FBS) and penicillin (100 μg/mL)/streptomycin (100 μg/mL) at 37 °C with 5% CO_2_ in a humidified environment. The medium was refreshed every two days. For the H_2_O_2–_induced neurotoxicity testing, SH-SY5Y cells in DMEM with low serum content (1% FBS) were seeded in 6-well or 96-well plates at a density of 1 × 10^5^ cells/mL for 24 h before further experiments.

Cell viability was assessed by the 3-(4,5-dimethylthiazol-2-yl)-2.5-diphenyltetrazolium bromide (MTT) assay based on previous protocol [31,32]. Here, 10 μL of MTT solution (5 mg/mL) was added to each well after treatment. Plates were incubated at 37 °C for 4 h in a humidified incubator. Subsequently, 100 μL of the solvating solution (0.01 N HCl in 10% SDS solution) was then added to each well, followed by incubation for 16–20 h. The absorbance of the samples was determined at a wavelength of 570 nm with 655 nm as a reference wavelength.

Viable cells were visualized by the fluorescein formed from FDA by esterase activity in viable cells. Non-viable cells were analyzed by PI staining, which only penetrates the membranes of dead cells. Then, the cells were examined after incubation with 10 μg/mL of FDA and 5 μg/mL of PI for 15 min. Images were acquired using UV light microscopy and these were compared with those taken under phase-contrast microscopy.

### 3.6. Statistical Analysis

Results were expressed as mean ± SEM. Differences among groups were compared by analysis of variance (ANOVA) followed by Dunnett’s or Tukey’s test. *p* < 0.05 was considered as statistically significant.

## 4. Conclusions

A new cyclopentenone, 5-hydroxycyclopenicillone (**1**) was isolated together with three known compounds (**2**–**4**) from the sponge-derived fungus, *Trichoderma* sp. HPQJ-34. These molecules were obtained at purified yields of about 0.01%, 0.03%, 0.04%, and 0.06%, respectively, from the dry EtOAc extract. Compound **1** is a new structural analogue of cyclopenicillone (**5**), which has only been reported once previously in the literature, thus this investigation expands the research available on a rare group of natural products. Compound **1** was shown to exhibit moderate in vitro anti-oxidative, free radical scavenging and anti-Aβ fibrillization activities, along with neuroprotective effects. Accordingly, 5-hydroxycyclopenicillone and structurally related molecules may be of interest to neuropharmacology research and anti-AD drug discovery programs. However, further studies are necessary to validate these results in vivo and to examine the penetration of these molecules into brain tissue.

## Figures and Tables

**Table 1:** The ^1^H-NMR (500 MHz) and ^13^C-NMR (125 MHz) data of compound **1** (in CD_3_OD).

Position	*δ*_C_, Type	*δ*_H_, Mult. (*J* in Hz)	^1^H–^1^H COSY	HMBC	NOESY
1	210.4, C				
2	135.8, C				
3	171.7, C				
4	75.3, CH	4.49, d (1.0)		3	7, 2′
5	74.5, C				
6	8.4, CH_3_	1.75, d (1.0)		1, 2, 3	1′
7	23.1, CH_3_	1.23, s		1, 4, 5	4
1′	69.4, CH	4.72, dd (8.6, 4.8)	2′	2, 3, 4, 2′, 3′	6, 2′, 3′
2′	36.6, CH_2_	1.86, dtd (14.2, 8.6, 5.5), 1.76, m	1′, 3′	3, 1′, 3′, 4′	4, 1′
3′	29.8, CH_2_	2.12, m	2′, 4′	1′, 5′	
4′	131.6, CH	5.49, m	3′, 5′		3′
5′	126.7, CH	5.48, m	4′, 6′	3′	6′
6′	18.1, CH_3_	1.64, d (4.8)	5′	4′, 5′	5′

## Supplementary Materials

The following are available online at www.mdpi.com/link/1660-3997/15/8/260/s1, Figure S1: HRESIMS for compound **1**, Figure S2: ^1^H NMR spectrum of compound **1**, Figure S3: ^13^C NMR spectrum of compound **1**, Figure S4: COSY spectrum of compound **1**, Figure S5: HSQC spectrum of compound **1**, Figure S6: HMBC spectrum of compound **1**, Figure S7: NOESY spectrum of compound **1**. Text S8: ITS rDNA gene sequence from strain HPQJ-34, Text S9: Computational molecular model coordinates for an energy minimized conformer of compound **1**, Text S10: Computational molecular model coordinates for an energy minimized conformer of compound **5**, Table S11: Comparison of the NMR data of compound 1 and 5.
