# Supplementary material for: 5-Hydroxycyclopenicillone, a New β-Amyloid Fibrillization Inhibitor from a Sponge-Derived Fungus Trichoderma sp. HPQJ-34
Source: Mar Drugs. 2017 Aug 19;15(8):260. doi: 10.3390/md15080260 (PMC5577614; doi:10.3390/md15080260)
Supplement: Supplementary file 1 [file marinedrugs-15-00260-s001.pdf]

Supplementary Materials: 5-Hydroxycyclopenicillone, a New β-Amyloid Fibrillization Inhibitor from a Sponge-Derived Fungus *Trichoderma* sp. HPQJ-34

Fang Fang, Jiaying Zhao, Lijian Ding, Chunhui Huang, C. Benjamin Naman, Shan He, Bin Wu, Peng Zhu, Qijun Luo, William H. Gerwick, Xiaojun Yan, Qinwen Wang, Zaijun Zhang and Wei Cui

List of Supplementary Materials:

Figure S1. HRESIMS for compound **1**.

Figure S2. ^{1}H NMR spectrum (CD_3OD, 500 MHz) of compound **1**.

Figure S3. ^{13}C NMR spectrum (CD_3OD, 125 MHz) of compound **1**.

Figure S4. COSY spectrum (CD_3OD, 500 MHz) of compound **1**.

Figure S5. HSQC spectrum (CD3OD, 500 MHz ^{1}H and 125 MHz ^{13}C) of compound **1**.

Figure S6. HMBC spectrum (CD3OD, 500 MHz ^{1}H and 125 MHz ^{13}C) of compound **1**.

Figure S7. NOESY spectrum (CD_3OD, 500 MHz) of compound **1**.

Text S8. ITS rDNA gene sequence from strain HPQJ-34.

Text S9. Computational molecular model coordinates for an energy minimized conformer of compound **1**.

Text S10. Computational molecular model coordinates for an energy minimized conformer of compound **5**.

Table S11. Comparison of the NMR data of compound **1** and **5**.

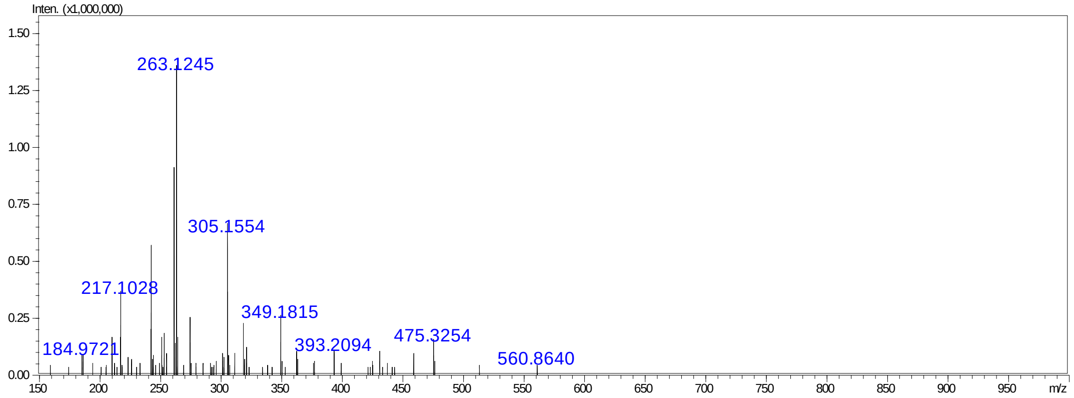

Figure S1. HRESIMS for compound **1**.

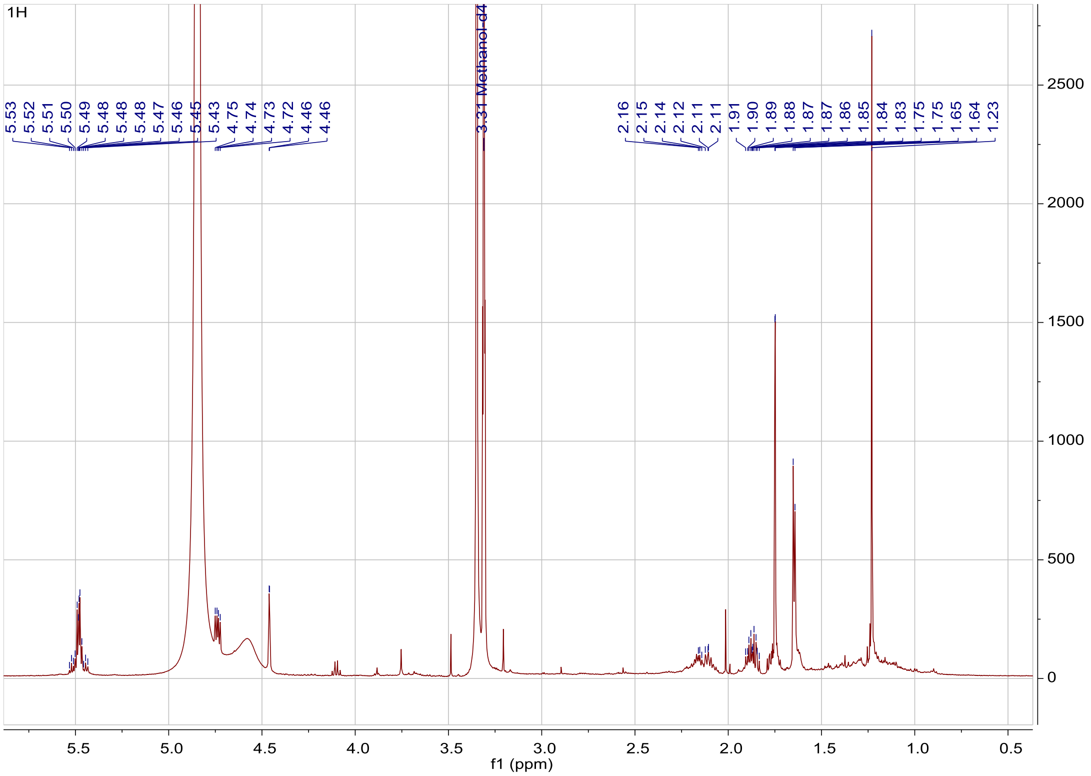

Figure S2. ^{1}H NMR spectrum (CD_3OD, 500 MHz) of compound **1**.

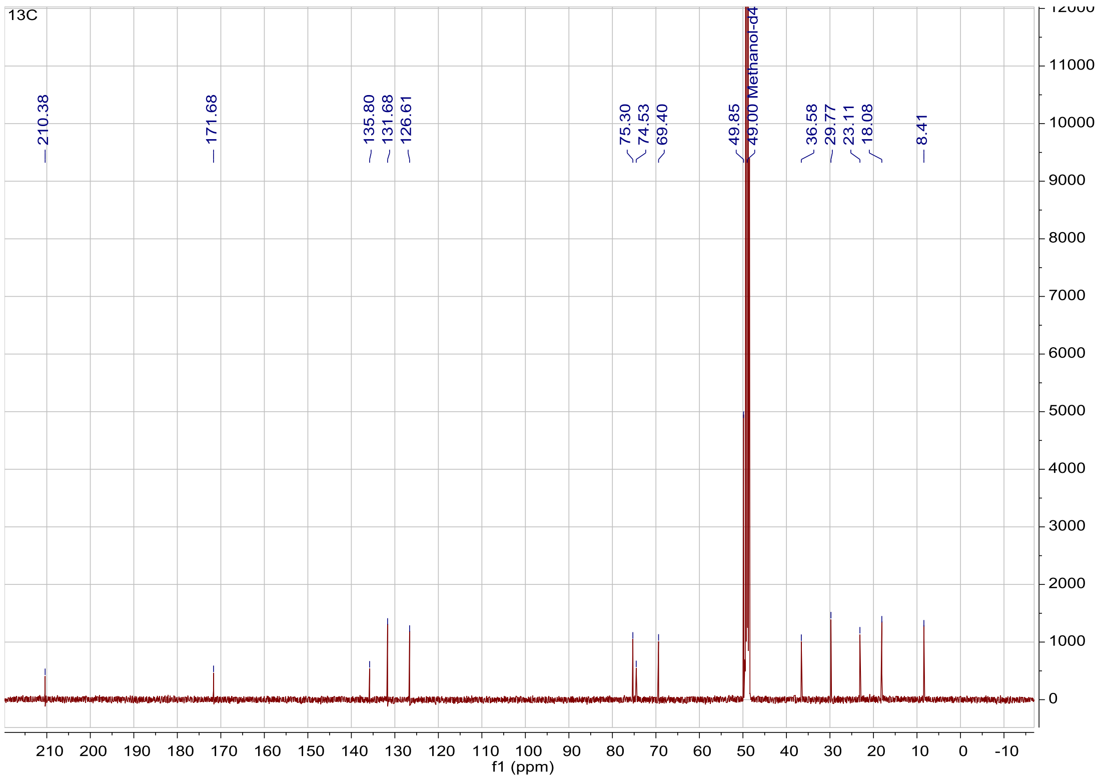

Figure S3. ^{13}C NMR spectrum (CD_3OD, 125 MHz) of compound **1**.

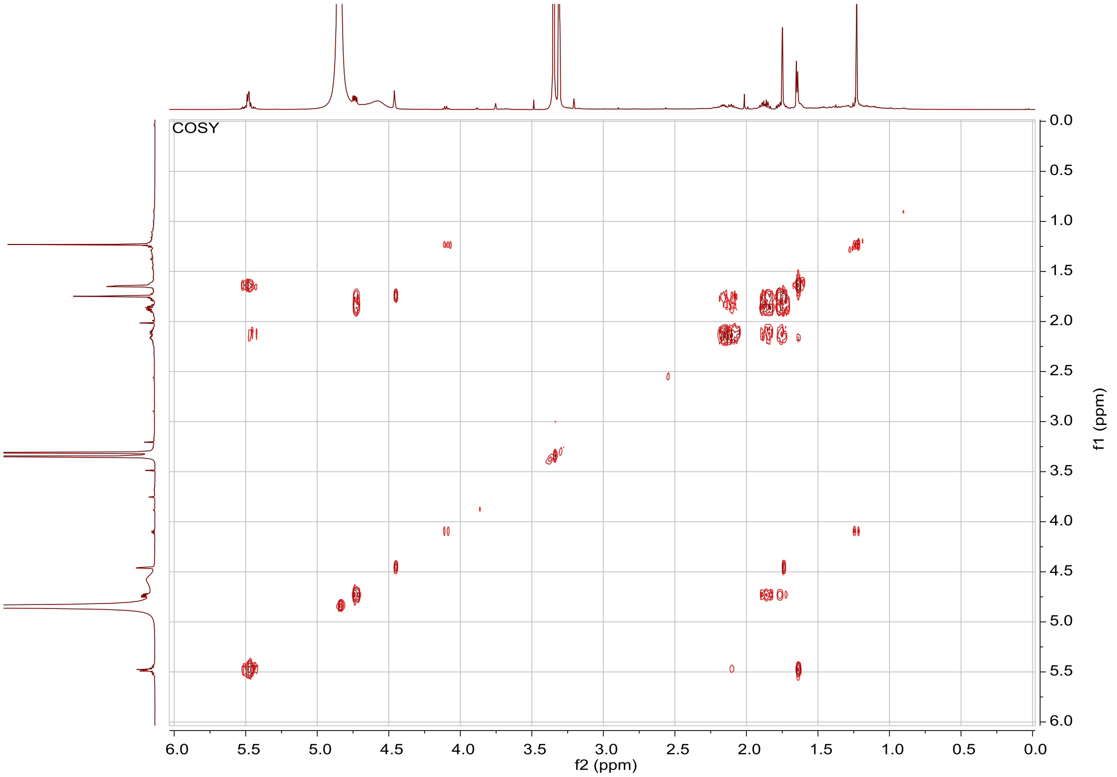

Figure S4. COSY spectrum (CD_3OD, 500 MHz) of compound **1**.

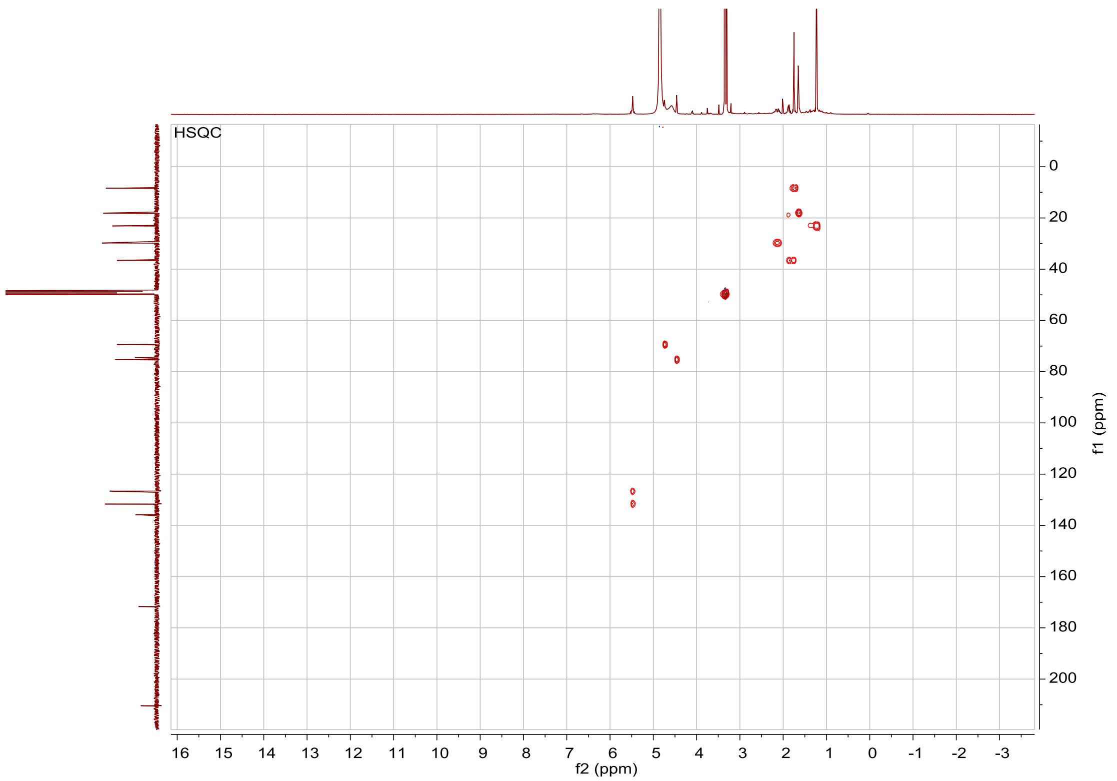

Figure S5. HSQC spectrum (CD$_3$OD, 500 MHz ^{1}H and 125 MHz ^{13}C) of compound **1**.

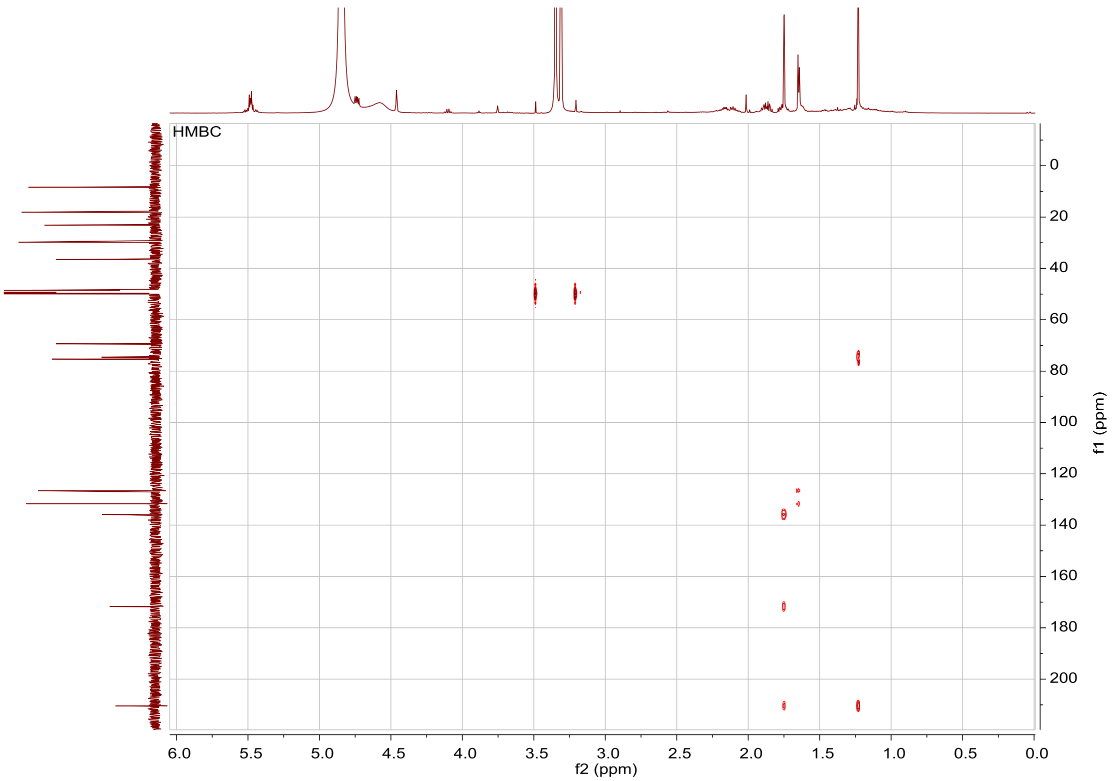

Figure S6. HMBC spectrum (CD$_3$OD, 500 MHz ^{1}H and 125 MHz ^{13}C) of compound **1**.

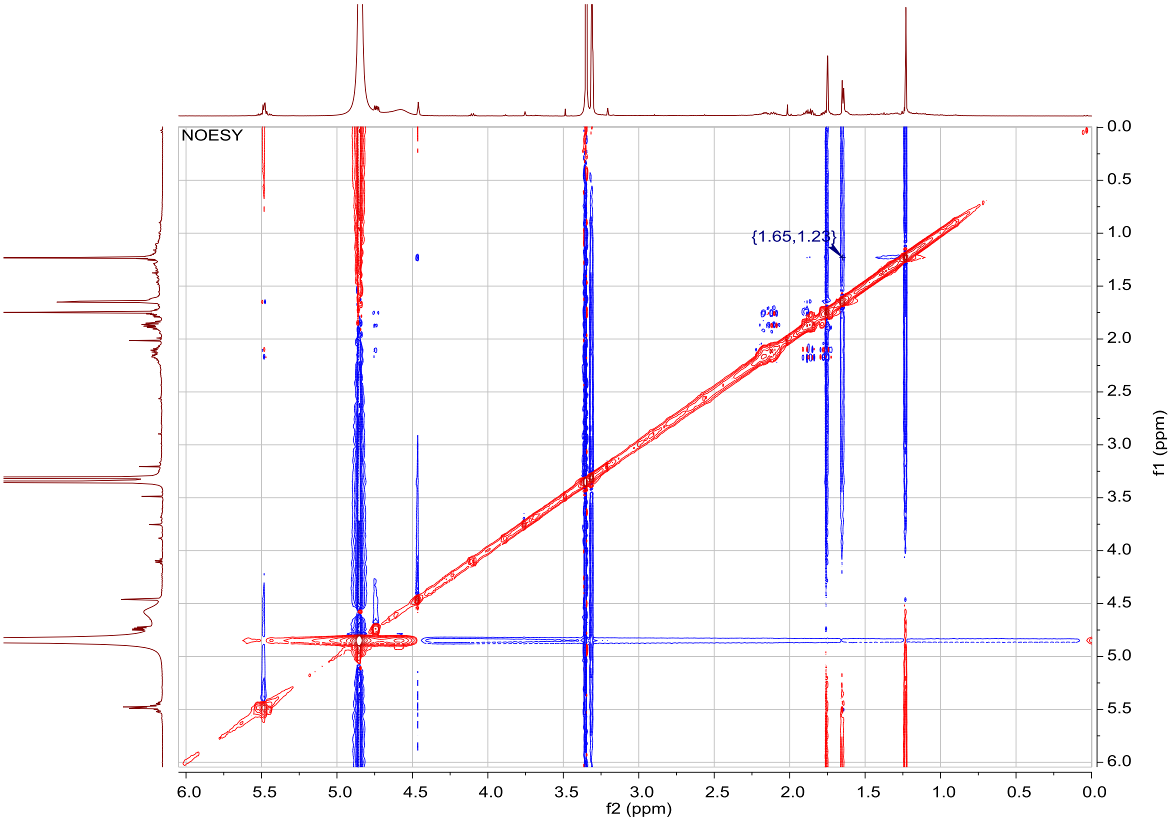

Figure S7. NOESY spectrum (CD3OD, 500 MHz) of compound **1**.

Text S8. ITS rDNA gene sequence from strain HPQJ-34.

TCCGTAGGTGGAACCTGCGGAGGGATCATTACCGAGTTTACAACTCCCAAACCC
CAATGTGAACGTTACCAATCTGTTGCCTCGGCGGGATTCTCTTGCCCCGGGCGC
GTCGCAGCCCCGGATCCCATGGCGCCCGCCGGAGGACCAACTCCAAACTCTTTT
TTCTCTCCGTCGCGGCTCCCGTCGCGGCTCTGTTTTATTTTTGCTCTGAGCCTTTC
TCGGCGACCCTAGCGGGCGTCTCGAAAATGAATCAAAACTTTCAACAACGGATC
TCTTGGTTCTGGCATCGATGAAGAACGCAGCGAAATGCGATAAGTAATGTGAAT
TGCAGAATTCAGTGAATCATCGAATCTTTGAACGCACATTGCGCCCGCCAGTAT
TCTGGCGGGCATGCCTGTCCGAGCGTCATTTCAACCCTCGAACCCCTCCGGGGG
GTCGGCGTTGGGGATCGGCCCCTCACCGGGCCGCCCCCGAAATACAGTGGCGGT
CTCGCCGCAGCCTCTCATGCGCAGTAGTTTGCACACTCGCACCGGGAGCGCGGC
GCGGCCACAGCCGTAAAACACCCCAAATTTTCTGAAATGTTGACCTCGGATCAG
G T A G G A A T A C C C G C T G A A C T T A A G C A T A T C A A T A A G C G G A A G A A

Text S9. Computational molecular model coordinates for an energy minimized conformer of compound **1**.

```
HEADER
REMARK Spartan '10 exported M0001
HETATM    1  C   UNK  0001      -1.637  -1.053   0.131
HETATM    2  C   UNK  0001      -0.687  -0.037  -0.488
HETATM    3  C   UNK  0001      -1.230   1.170  -0.597
HETATM    4  C   UNK  0001      -2.600   1.142  -0.015
HETATM    5  C   UNK  0001      -2.773  -0.191   0.725
HETATM    6  O   UNK  0001      -3.403   2.017  -0.086
HETATM    7  C   UNK  0001      -0.694   2.436  -1.193
HETATM    8  O   UNK  0001      -2.193  -1.884  -0.854
HETATM    9  C   UNK  0001      -2.587   0.056   2.220
HETATM   10  C   UNK  0001       0.690  -0.465  -0.926
HETATM   11  C   UNK  0001       1.707  -0.333   0.213
HETATM   12  C   UNK  0001       3.132  -0.750  -0.181
HETATM   13  C   UNK  0001       4.099  -0.585   0.962
HETATM   14  O   UNK  0001       0.571  -1.806  -1.366
HETATM   15  C   UNK  0001       5.146   0.218   1.041
HETATM   16  C   UNK  0001       5.667   1.184   0.012
HETATM   17  H   UNK  0001      -1.148  -1.658   0.887
HETATM   18  H   UNK  0001       0.229   2.281  -1.740
HETATM   19  H   UNK  0001      -1.429   2.863  -1.866
HETATM   20  H   UNK  0001      -0.509   3.173  -0.418
HETATM   21  H   UNK  0001      -1.484  -2.303  -1.327
HETATM   22  H   UNK  0001      -3.353   0.738   2.567
HETATM   23  H   UNK  0001      -2.690  -0.881   2.754
HETATM   24  H   UNK  0001      -1.614   0.483   2.439
HETATM   25  H   UNK  0001       1.009   0.153  -1.760
HETATM   26  H   UNK  0001       1.718   0.702   0.541
HETATM   27  H   UNK  0001       1.371  -0.931   1.055
HETATM   28  H   UNK  0001       3.133  -1.798  -0.475
HETATM   29  H   UNK  0001       3.451  -0.179  -1.047
HETATM   30  H   UNK  0001       3.886  -1.208   1.818
HETATM   31  H   UNK  0001       1.364  -2.080  -1.806
HETATM   32  H   UNK  0001       5.716   0.192   1.956
HETATM   33  H   UNK  0001       6.685   0.927  -0.268
HETATM   34  H   UNK  0001       5.070   1.210  -0.890
HETATM   35  H   UNK  0001       5.696   2.191   0.421
HETATM   36  O   UNK  0001      -4.031  -0.751   0.557
```

```
HETATM   37  H    UNK  0001        -4.044  -1.232  -0.261
CONECT    1   17    2    5    8
CONECT    2    1    3   10
CONECT    3    2    4    7
CONECT    4    3    5    6
CONECT    5    4    1    9   36
CONECT    6    4
CONECT    7    3   18   19   20
CONECT    8    1   21
CONECT    9    5   22   23   24
CONECT   10    2   11   14   25
CONECT   11   10   12   26   27
CONECT   12   11   13   28   29
CONECT   13   12   15   30
CONECT   14   10   31
CONECT   15   13   16   32
CONECT   16   15   33   34   35
CONECT   17    1
CONECT   18    7
CONECT   19    7
CONECT   20    7
CONECT   21    8
CONECT   22    9
CONECT   23    9
CONECT   24    9
CONECT   25   10
CONECT   26   11
CONECT   27   11
CONECT   28   12
CONECT   29   12
CONECT   30   13
CONECT   31   14
CONECT   32   15
CONECT   33   16
CONECT   34   16
CONECT   35   16
CONECT   36   37    5
CONECT   37   36
END
```

Text S10. Computational molecular model coordinates for an energy minimized conformer of compound **5**.

```
HEADER
REMARK Spartan '10 exported M0001
HETATM    1  C   UNK  0001    -1.879  -1.202   0.182
HETATM    2  C   UNK  0001    -0.933  -0.161  -0.408
HETATM    3  C   UNK  0001    -1.443   1.067  -0.395
HETATM    4  C   UNK  0001    -2.783   1.036   0.240
HETATM    5  C   UNK  0001    -3.124  -0.403   0.589
HETATM    6  O   UNK  0001    -3.467   1.989   0.459
HETATM    7  C   UNK  0001    -0.868   2.370  -0.866
HETATM    8  O   UNK  0001    -2.252  -2.186  -0.741
HETATM    9  C   UNK  0001    -3.551  -0.554   2.049
HETATM   10  C   UNK  0001     0.456  -0.536  -0.868
HETATM   11  C   UNK  0001     1.495  -0.240   0.222
HETATM   12  C   UNK  0001     2.935  -0.575  -0.192
HETATM   13  C   UNK  0001     3.920  -0.255   0.902
HETATM   14  O   UNK  0001     0.446  -1.912  -1.198
HETATM   15  C   UNK  0001     4.901   0.632   0.889
HETATM   16  C   UNK  0001     5.310   1.561  -0.221
HETATM   17  H   UNK  0001    -1.413  -1.669   1.048
HETATM   18  H   UNK  0001    -3.938  -0.713  -0.058
HETATM   19  H   UNK  0001     0.037   2.238  -1.448
HETATM   20  H   UNK  0001    -1.595   2.896  -1.473
HETATM   21  H   UNK  0001    -0.638   3.012  -0.021
HETATM   22  H   UNK  0001    -1.463  -2.594  -1.074
HETATM   23  H   UNK  0001    -4.402   0.082   2.258
HETATM   24  H   UNK  0001    -3.826  -1.581   2.261
HETATM   25  H   UNK  0001    -2.749  -0.272   2.725
HETATM   26  H   UNK  0001     0.701   0.038  -1.757
HETATM   27  H   UNK  0001     1.436   0.812   0.480
HETATM   28  H   UNK  0001     1.230  -0.801   1.114
HETATM   29  H   UNK  0001     3.011  -1.639  -0.412
HETATM   30  H   UNK  0001     3.183  -0.046  -1.106
HETATM   31  H   UNK  0001     3.784  -0.829   1.805
HETATM   32  H   UNK  0001     1.235  -2.141  -1.668
HETATM   33  H   UNK  0001     5.498   0.715   1.783
HETATM   34  H   UNK  0001     6.337   1.367  -0.519
HETATM   35  H   UNK  0001     4.687   1.475  -1.102
HETATM   36  H   UNK  0001     5.269   2.593   0.116
```

```
CONECT    1   17    2    5    8
CONECT    2    1    3   10
CONECT    3    2    4    7
CONECT    4    3    5    6
CONECT    5    4    1    9   18
CONECT    6    4
CONECT    7    3   19   20   21
CONECT    8    1   22
CONECT    9    5   23   24   25
CONECT   10    2   11   14   26
CONECT   11   10   12   27   28
CONECT   12   11   13   29   30
CONECT   13   12   15   31
CONECT   14   10   32
CONECT   15   13   16   33
CONECT   16   15   34   35   36
CONECT   17    1
CONECT   18    5
CONECT   19    7
CONECT   20    7
CONECT   21    7
CONECT   22    8
CONECT   23    9
CONECT   24    9
CONECT   25    9
CONECT   26   10
CONECT   27   11
CONECT   28   11
CONECT   29   12
CONECT   30   12
CONECT   31   13
CONECT   32   14
CONECT   33   15
CONECT   34   16
CONECT   35   16
CONECT   36   16
END
```

Table S11. Comparison of the NMR data of compound **1**[a] and **5**[b]

Position	δ_C, type.	δ_H, mult. (J in Hz)	δ_C, type.	δ_H, mult. (J in Hz)
		**1**		**5**
1	210.4, C		210.3, C	
2	135.8, C		136.4, C	
3	171.7, C		173.5, C	
4	75.3, CH	4.49, d (1.0)	77.6, CH	4.46, br s
5	74.5, C		50.9, CH	2.18, q (7.8)
6	8.4, CH_3	1.75, d (1.0)	8.3, CH_3	1.73, br s
7	23.1, CH_3	1.23, s	13.8, CH_3	1.16, d (7.8)
1′	69.4, CH	4.72, dd (8.6, 4.8)	69.7, CH	4.75, dd (8.4, 4.2)
2′	36.6, CH_2	1.86, dtd (14.2, 8.6, 5.5), 1.76, m	36.9, CH_2	1.85, m, 1.64, m
3′	29.8, CH_2	2.12, m	29.8, CH_2	2.18, m, 2.10 m
4′	131.6, CH	5.49, m	131.7, CH	5.49, m
5′	126.7, CH	5.48, m	126.6, CH	5.48, m
6′	18.1, CH_3	1.64, d (4.8)	18.1, CH_3	1.63, d (6.6)

[a] Isolated in this study; "NMR spectra were obtained at 500 MHz for ^{1}H NMR and 125 MHz for ^{13}C NMR on a Bruker AVANCE-500 spectrometer. Chemical shifts (δ) are referenced to the residual solvent peaks of CD_3OD (δ_H 3.31 and δ_C 49.0) and given in ppm".

[b] Not isolated in this study; "… in CD_3OD. Data were recorded on a Bruker Avance 600 spectrometer; chemical shifts (δ) are given in parts per million with references to the center peak of CD_3OD with δ 3.30 for ^{1}H and δ 49.0 for ^{13}C." from manuscript reference 19 [Lin, S.; Shi, T.; Chen, K. Y.; Zhang, Z. X.; Shan, L.; Shen, Y. H.; Zhang, W. D. Cyclopenicillone, a unique cyclopentenone from the cultures of *Penicillium decumbens*. *Chem. Commun.* **2011**, *47*, 10413–10418.].
